# Inhibitory Effects of Donkey Hide Gelatin on DNCB-Induced Atopic Dermatitis in NC/Nga Mice

**DOI:** 10.3389/fphar.2022.896450

**Published:** 2022-05-26

**Authors:** Ju Hyun Lee, Linsha Dong, Hyeon Min Noh, Sung-Gu Park, Seung-Hyung Kim, Eun Heui Jo, Dong-Sung Lee, Min Cheol Park

**Affiliations:** ^1^ Department of Korean Medicine Ophthalmology and Otolaryngology and Dermatology, Wonkwang University Korean Medicine Hospital, Iksan, South Korea; ^2^ College of Pharmacy, Chosun University, Gwangjuu, South Korea; ^3^ Weedahm Korean Medicine Hospital, Gangnam, South Korea; ^4^ Resam Korean Medicine Hospital, Gangnam, South Korea; ^5^ Institute of Traditional Medicine and Bioscience, Daejeon University, Daejeon, South Korea; ^6^ Department of Acupuncture and Moxibustion, Wonkwang University Korean Medicine Hospital and Research Center of Traditional Korean Medicine, Wonkwang University, Iksan, South Korea; ^7^ Department of Korean Medicine Ophthalmology and Otolaryngology and Dermatology, Wonkwang University Korean Medicine Hospital and Research Center of Traditional Korean Medicine, Wonkwang University, Iksan, Korea

**Keywords:** allergy, atopic dermatitis, donkey hide gelatin, immunology, hacat cell

## Abstract

The increase of atopic dermatitis has led to higher socio-economic cost and raised a need for alternative medicine as novel therapeutic agents. In this study, we aimed to evaluate the inhibitory effects of Donkey Hide Gelatin (DHG) water extract on DNCB-induced atopic dermatitis in NC/Nga mice and on tumor necrosis factor (TNF)-α/interferon (IFN)-γ-treated keratinocytes and to investigate its underlying molecular mechanisms. NC/Nga mice were induced by DNCB, administered Dexamethasone (3 mg/kg) or DHG water extracts (100–400 mg/kg) for 3 weeks. The skin symptom score, serum IgE and immune cells were measured, the ALN, spleen and dorsal skin tissue were extracted for FACS, quantitative real-time PCR and histology analysis. *In vitro*, HaCaT cells were induced by TNF-α/IFN-γ, the levels of pro-inflammatory cytokines and chemokines and its underlying mechanism were measured by ELISA and Western blot. As a result, DHG groups showed a significant decrease in the skin symptom score and the immune cell absolute number. It also showed a marked reduction of allergic and the levels of neutrophils and eosinophils in histology analysis. In TNF-α/IFN-γ induced HaCaT cells, DHG showed inhibition effects on IL-6, IL-8, TARC and RANTES, it also downregulated the expression of ICAM-1 and COX-2, up-regulated the expression of Filaggrin. Furthermore, DHG suppressed the activation of NF-κB and mitogen-activated protein kinases (MAPK) signaling pathway induced by TNF-α/IFN-γ. Taken together, DHG maybe a potential therapeutic agent or supplement for skin inflammatory disease such as atopic dermatitis.

## Introduction

Atopic dermatitis (AD) is a chronic inflammatory skin disease characterized by pruritis skin lesions (Lawrence et al., 2014). While the main cause and pathogenesis of atopic dermatitis remain unclear, it is likely that a complex interaction of multiple factors, including allergen and microbial exposure, immune dysfunction, and impaired skin barrier function plays an important role in development of AD (Carroll et al., 2005). The methods used in the treatment of atopic dermatitis according to the cause and severity of symptoms are topical anti-inflammatory therapy, phototherapy, avoidance strategies, emollient therapy (skin care), and alternative medicine, among which the treatment most generally recommended is the topical steroid treatment (Arellano et al., 2007; Wollenberg et al., 2018a; Wollenberg et al., 2018b). However, a long-term steroid use may induce side effects such as steroid rosacea, acne, atrophy, perioral dermatitis, and telangiectasia. The steroid treatment efficiency diminishes in the patients with poor reactivity to steroid drugs, while the potential recurrence of atopic dermatitis increases (Fukaya, 2000; Atherton, 2003). The prevalence of atopic dermatitis has steadily increased for several centuries, and at present, approximately 15–20% of global population is affected by the condition (Bantz et al., 2014). The higher socio-economic cost due to AD has gradually increased the need for novel therapeutic agents (Suh et al., 2007). This led to an ever-increasing interest in complementary and alternative medicine (Park et al., 2015; Park et al., 2019).

Donkey Hide Gelatin (DHG; *Colla corii asini*) is produced using a method where the skin of *Equus asinus* L. is heated with water for extraction. In general, DHG is a yellowish-brown solid that turns into a colloid in an aqueous solution, while it enters a sol state upon heating and a gel state upon cooling. The main ingredients consist mostly of gelatin and collagen, and in some cases, protein (Chen et al., 1991). The known effects of DHG include ‘hematopoietic effect (Wu et al., 2007; Zhang et al., 2018)', ‘anti-oxidant/anti-aging effect (Wang et al., 2012; Zhang et al., 2012)', and ‘anti-bacterial effect (Park et al., 2017)'. Recently, DHG was reported to exhibit anti-allergic effects based on the regulation of immune substances such as promoting the macrophage and IFN-γ activities while reducing the IL-4 level (Zhang et al., 2011). However, there is a general lack of studies proving the effects of DHG on atopic dermatitis, with the exception of an experimental study reporting the effects of DHG that could inhibit Dfb-induced AD-skin-like symptoms (Kang et al., 2022) And an alternative medicine containing DHG called Jagamcho-tang. it could inhibit the Th2-related cytokine production for reducing the IgE production, thereby improving the allergic inflammatory response to OVA (Noh et al., 2017). 2,4-dinitrochlorobenzene (DNCB), an electrophilic and cytotoxic benzene derivative, induces stable clinical AD-like skin diseases in NC/Nga mice. It was the first to prove the effect of DHG in DNCB-induced AD-like skin diseases. In this study, we aimed to evaluate inhibitory effects of DHG water extract on atopic dermatitis in DNCB-induced NC/Nga mice and tumor necrosis factor (TNF)-α/interferon (IFN)-γ-treated keratinocytes.

## Materials and Methods

### Animals

**FIGURE 1:**
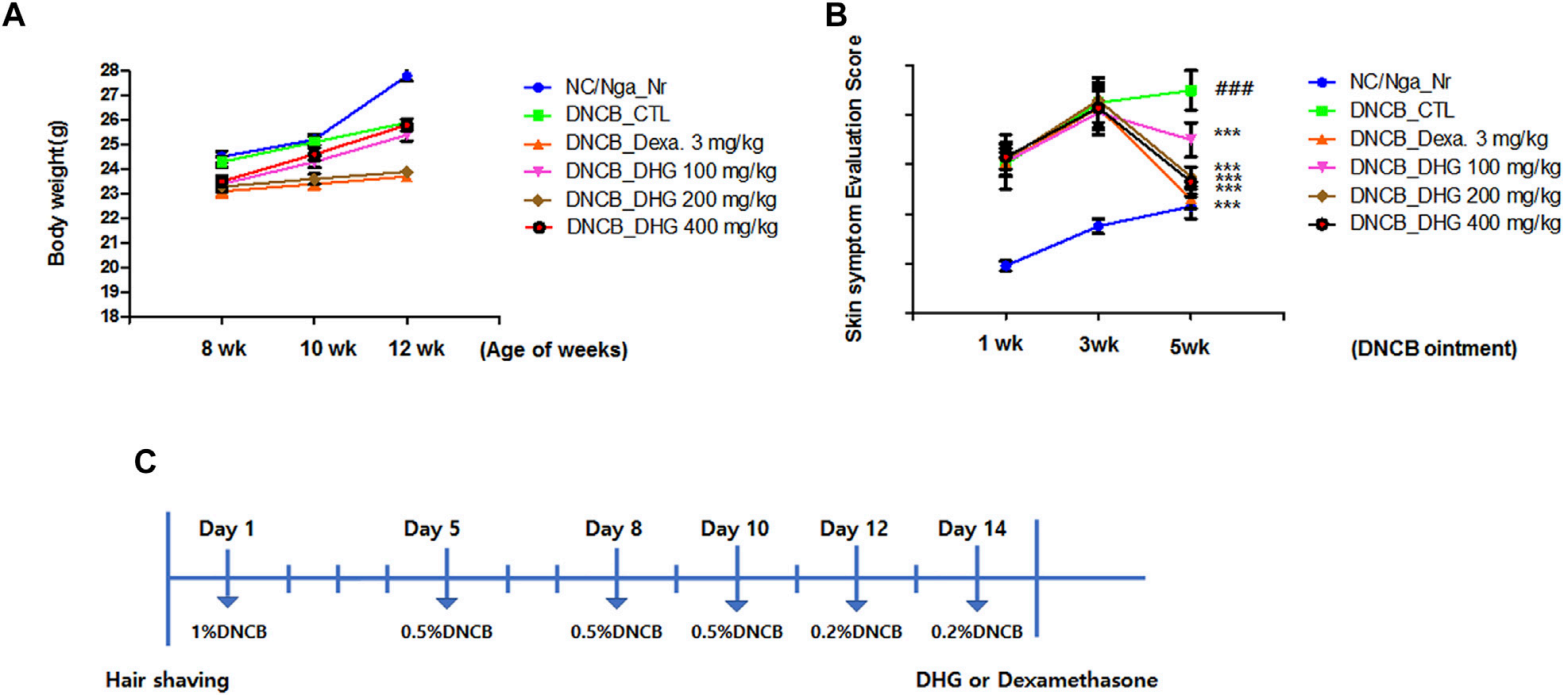
Changes in body weight **(A)** and skin features and severity **(B)** in NC/Nga mice (total *n* = 6/group). Schematic diagram of the experimental protocol in NC/Nga mice **(C)**.

Male NC/Nga mice (7 weeks of age) were purchased from the OrientBio (Seongnam-si, South Korea). This animal experiment was conducted following the approval of the Institutional Animal Care and Use Committee at Daejeon University (Approval No, DJUARB 2019-041). Animal experiments were performed in accordance with the established institutional guidelines regarding animal care and use and the Guide for Care and Use of Laboratory Animals (National Research Council of United States , 1996). The mice were given a period of 1-week adaptation to the conditions of temperature 22 ± 2°C, humidity 55 ± 15%, and 12 h light-dark cycle. NC/Nga mice were left to rest for 24 h, after the shaving from the lower end of the ear to the upper end of the tail. Next, 200 μL of 1% DNCB (dinitrochlorobenzene) solution (acetone: olive oil = 3 : 1) was applied to the shaved area in mice, and after 4 days, the second application using 0.5% DNCB solution was carried out. From day 7 after the first application, the third application using 0.5% solution was carried out. And in the next week, we using 0.5, 0.2 and 0.2% DNCB solution one time every 2 days, for inducing atopic dermatitis.(Figure 1C).

### Skin Symptom Evaluation of the Induced Atopic Dermatitis

The skin symptom evaluation of the NC/Nga mice with induced atopic dermatitis was carried out in 1-week interval based on three criteria. The investigators examined each mice to record the level of 1) erythema/hemorrhage, 2) scaling/dryness, and 3) excoriation/erosion, as the score of 0 (none), 1 (mild), 2 (moderate), and 3 (severe), then by adding the scores up, defined the skin symptom evaluation score.

### Sample Preparation

DHG used in this study was supplied from Kherb Research Center, Nong-Lim. Voucher specimens of DHG have been deposited at Nong-Lim (NL023). 30 g of DHG (Donkey Hide Gelatin) was added to 1,040 ml of purified water, and for extraction, a pneumatic extractor (KYUNGSEO E&P, Korea) was used, then to concentrate the filtrate, a vacuum distillation unit (BuchiB-480, Switzerland) was used. The enriched filtrate was completely dehydrated using the freeze dryer (EyelaFDU-540, Japan), then stored in a freezer (-84°C) for subsequent use. Through this process, the final 17.63 g of freeze-dried powder was isolated and purified from 30 g of DHG. For the *in vitro* study, the extract powder that resulted from drying process was dissolved in distilled water.

### Intervention

To the NC/Nga mice with atopic dermatitis induced by DNCB, the trial drug was orally administered once a day at a fixed time for 3 weeks. Dexamethasone (3 mg/kg) (Sigma-Aldrich, United States ) was administered to the positive control group (DNCB_Dexa group), and the DHG water extracts (100 mg/kg, 200 mg/kg, and 400 mg/kg) were administered to the experimental groups. The negative control group (DNCB_CTL group) did not take any treatment after inducing atopic dermatitis. A total of thirty NC/Nga mice were divided into six groups (*n* = 5) as follows: 1) NC/Nga_Nr group, 2) DNCB_CTL group, 3) DNCB_Dexa group, 4) DNCB_DHG 100 mg/kg group, 5) DNCB_DHG 200 mg/kg group, 6) DNCB_DHG 400 mg/kg group. The experimental group and the negative control group were statistically compared and analyzed. In addition, to clearly measure the efficacy, the result values of the experimental group were compared with the positive control group.

### Fluorescence-Activated Cell Sorting Analysis of ALN, Spleen, and Dorsal Skin Tissue

For the isolated, infiltrated cells of ALN, spleen, and dorsal skin tissue, the total cell count was measured, and after adjusting the cell count for all tissues to 5 × 10^5^, the immunofluorescence staining was carried out at 4°C. Next, anti-CD3, anti-CD11, anti-CD4, anti-CD8, anti-CD23, anti-CD69, anti-Gr-1, and anti-B220 were added to each, for 30 min reaction on ice. At the completion of reaction, PBS was used for the washing for three or more times, and using the Cell Quest program of the flow cytometry, the cell count was analyzed as percentage (%). To these values, the total cell count was applied, to estimate the absolute number for each tissue.

### Cell Culture: DHG Treatment and Stimulation

HaCaT cell lines originating from human keratinocytes were purchased from the Korea cell line bank (Seoul, Korea). HaCaT cells were cultured in Dulbecco’s Modified Eagle Medium (DMEM) with 10% fetal bovine serum (FBS; Gibco-BRL, Grand Island, NY, United States ). Cytotoxicity was measured by MTT assay, HaCaT cells were treated with 0–400 μg/ml DHG for 24 h, 5 mg/ml 3-[4,5-dimethylthiazol-2-yl]-2,5-diphenyltetrazolium bromide (MTT) was added to 1 ml of cell suspension and incubated for 1 h. The formazan formed was dissolved in DMSO, and optical density was measured at 540 nm. Inflammatory responses were stimulated in HaCaT cells by treatment with 20 ng/ml of TNF-α/IFN-γ (each 10 ng/ml) (Sigma-Aldrich). Next, 50–200 μg/ml of the DHG extract was supplemented to the cell culture. Samples were first incubated for 3 h, and the cells were subsequently induced with 20 ng/ml of TNF-α and IFN-γ for 24 h.

### Measurement of IL-6, IL-8, TARC and RANTES in TNF-α/IFN-γ induced HaCaT

The cells were seeded at densities of 5.0 × 10^5^ cells/mL in 24 well plate. The cells were incubated for 3 h with DHG and then stimulated with TNF-α/IFN-γ for 24 h. After that the supernatant was collected and harvested to further evaluate IL-6, IL-8, TARC and RANTES secretions. They were evaluated using ELISA kit, according to the manufacturer’s instructions.

### Western Blot Analysis

HaCaT cells were pre-treated with DHG for 3 h, stimulated with TNF-α/IFN-γ for 24 h. Then, the cells were harvested and total protein was extracted using cell lysis buffer. Proteins were separated by electrophoresis on an SDS-polyacrylamide gel, and transferred to NC membranes. Nonspecific binding was blocked by treatment with 5% skim milk dissolved in TBST for 45 min at room temperature. The membranes were then incubated overnight in 4°C with a 1:1,000 dilution of primary antibodies. After washing 3 times with TBST buffer, the membranes were incubated with 1: 2000 dilution of horseradish peroxidase-conjugated secondary antibody for 1 h at room temperature. The membranes were analyzed using the ImageJ (NIH, Rockville, MD, United States )

### Isolation of Nuclear and Cytoplasmic Fractions

HaCaT cells were pre-treated with DHG for 3 h, and then stimulated with TNF-α/IFN-γ for 15 min. After the experiment, the cells were harvested, washed with 1 ml of ice-cold PBS, and centrifuged at ×500 g for 5 min. Nuclear and cytoplasmic protein fractions were extracted using Extraction Reagents kit (Caymen, Michigan, United States ), according to the manufacturer’s instructions.

### Th1/Th2 Cytokine Expression in Splenocytes

The levels of IL-4 (R and D system, United States ), IFN-γ (R&D system, United States ), IL-5 (R and D system, United States ) and IL-13 (R and D system, United States ) in the splenocyte culture supernatant, were measured using the enzyme-linked immuno-sorbent assay.

### Quantitative Real-Time PCR in Dorsal Skin Tissue

A certain amount of the resulting solution extracted from dorsal skin was used for RNA quantification, and the rest was used for cDNA synthesis using the PrimeScript™ RT reagent kit (TaKaRa, Shiga, Japan) and following the manufacturer’s instructions. The cDNA, and IL-5, IL-13, IL-31, TNF-α, CCR3, and glyceraldehyde-3 phosphate dehydrogenase (GAPDH) primer were each diluted to an adequate concentration, then mixed, and using the reagents; Power SYBR® Green PCR Master Mix and TaqMan® Gene Expression Master Mix (Applied Biosystems, CA, United States ), the analysis was carried out through the real-time polymerase chain reaction (real-time PCR) system (Applied Biosystems, CA, United States ). The mouse oligonucleotide sequence is as shown below (Table 1).

**TABLE 1:** Primer sequence.

Gene	Primer	Sequence
GAPDH	Forward	5′ TGA​AGC​AGG​CAT​CTG​AGG​G 3′
	Reverse	5′ CGA​AGG​TGG​AAG​AGT​GGG​AG 3′
IL-5	Forward	5′ AGC​CTA​ACC​CTG​TTG​GAG​GT 3′
	Reverse	5′ GTG​ATC​GGC​TTT​TCT​TGA​GC 3′
IL-13	Forward	5′ ATG​CCC​AAC​AAA​GCA​GAG​AC 3′
	Reverse	5′ TGA​GAG​AAC​CAG​GGA​GCT​GT 3′
IL-31	Forward	5′ TCA​GCA​GAC​GAA​TCA​ATA​CAG​C 3‘
	Reverse	5' TCG​CTC​AAC​ACT​TTG​ACT​TTC​T 3‘
TNF-α	Forward	5' TGG​GAG​GAA​AGG​GGT​CTA​AG 3'
	Reverse	5' ACC​TAC​GAC​GTG​GGC​TAC​AG 3'
CCR3	Forward	5 ‘AAA​GCT​GAT​ACC​AGA​GCA​CTG3'
	Reverse	5 ‘CCA​AGA​GGC​CCA​CAG​TGA​AC3'

### IgE, Neutrophils, Eosinophils, and Lymphocytes Level Measurement

At weeks 1, 2, and five of the experiment, approximately 100 μL of blood was collected from the eye of NC/Nga mice using a capillary tube, which was used to isolate 30 μL of serum through 20 min centrifugation at 6,500 rpm. The serum was stored in -80°C freezer, and for the measurement of serum IgE level, the enzyme-linked immuno-sorbent assay was carried out. Also, 0.5 ml of blood was collected from the heart of NC/Nga mice using a tube syringe treated with EDTA. The collected whole blood was sent to the Department of Clinical Pathology at Daejeon University for the measurement of total cell count of neutrophils, eosinophils, and lymphocytes in the blood.

### Histology Analysis

At the end of the experiment, the dorsal skin tissue was fixed in 10% formalin for 24 h. The fixed tissue was paraffin-formatted to be made into a block of 5 μm thickness. To discriminate the inflammatory epidermis, dermis, keratinocytes, and neutrophils/eosinophils from the mast cells, the hematoxyline and eosin (H&E) and toluidine blue staining assays were carried out, and the infiltration was examined using a light microscope (Nikon, Japan, ×200).

### Statistical Analysis

The result values for each group were given as mean ± standard deviation (SD), and the SPSS 11.0 software (IBM-SPSS Inc, Chicago, United States ) was used to carry out the one-way analysis of variance (ANOVA), followed by Duncan’s multiple comparison tests for testing the significance. Statistical significance was set at *p < 0*.05.

## Results

### The Effects of DHG in Body Weight and Skin Symptom Evaluation Score

To examine the changes in body weight, the weight of the animal was continuously measured for 5 weeks after the experiment started. The result indicated no notable change in body weight for 5 weeks in the DNCB_Dexa group, while all other experimental groups showed a slight increase in weight (Figure 1). Skin symptom evaluation score evaluation was carried out for 5 weeks after the application of DNCB, the DNCB_CTL group showed a significant increase in the score, compared to the NC/Nga-Nr group. The experimental groups and the DNCB_Dexa group showed a statistically significant decrease in the skin symptom evaluation score, compared to the DNCB_CTL group (Figure 1).

### The effects of DHG in ALN and Spleen and Dorsal Skin Tissue Immune Cell Absolute Number

The total cell counts of the ALN, spleen, and dorsal skin tissue was statistically significantly increased in the DNCB_CTL group, compared to the NC/Nga_Nr group. In contrast, the experimental groups and the DNCB_Dexa group showed a statistically significant reduction compared to the DNCB_CTL group. For ALN, the absolute number of CD3^+^, CD4^+^, CD4^+^/CD69^+^, and CD23^+^/B220^+^ showed a statistically significant increase in the DNCB_CTL group, whereas the DNCB_Dexa group and the experimental groups showed a statistically significantly reduced absolute number in a concentration-dependent manner. For spleen, the absolute number showed a decreasing trend as in the case of ALN, but without statistical significance. For dorsal skin tissue, a statistically significantly increased absolute number of CD4^+^ and Gr-1^+^/CD11b^+^ was shown in the DNCB_CTL group, whereas the DNCB_Dexa group and the experimental groups showed a statistically significantly decreased absolute number (Table.2).

**TABLE 2:** The effects of DHG on ALN and spleen and dorsal skin tissue immune cell absolute number.

Cell phenotypes in ALN and spleen and D-skin		NC/Nga-Nr	DNCB_CTL	DNCB_Dexa.3 mg/kg	DNCB_DHG 100 mg/kg	DNCB_DHG 200 mg/kg	DNCB_DHG 400 mg/kg
CD3^+^(×10^4^ cells)	ALN	26.68 ± 4.15	229.43 ± 37.77^###^	127.14 ± 20.04**	217.81 ± 9.03	186.83 ± 9.58*	144.92 ± 1.16*
CD4^+^(×10^4^ cells)		15.08 ± 3.02	132.10 ± 17.55^####^	75.09 ± 9.17**	115.53 ± 5.06	105.63 ± 12.35*	79.01 ± 0.45**
CD4^+^/CD69^+^(×10^4^ cells)		4.47 ± 0.41	90.30 ± 12.32^####^	20.87 ± 4.16***	31.01 ± 3.43***	29.20 ± 2.50***	21.92 ± 3.18***
CD23^+^/B220^+^(×10^4^ cells)		9.12 ± .0.40	120.96 ± 12.70^####^	36.93 ± 5.70***	86.49 ± 5.95*	70.13 ± 8.93	49.55 ± 3.11**
CD3^+^(×10^5^ cells)	Spleen	32.64 ± 4.62	46.98 ± 13.66	31.55 ± 8.31	40.08 ± 8.76	35.56 ± 4.30	25.39 ± 8.34
CD4^+^(×10^5^ cells)		12.56 ± 3.14	18.45 ± 3.42	12.48 ± 3.51	13.92 ± 1.72	12.65 ± 1.52	11.50 ± 3.60
CD8^+^(×10^5^ cells)		4.20 ± 0.51	7.68 ± 1.81	5.52 ± 1.08	7.96 ± 0.98	4.97 ± 0.84	3.51 ± 1.16
CD4^+^/CD69^+^(×10^5^ cells)		1.77 ± 0.52	2.51 ± 0.72	1.37 ± 0.34	1.68 ± 0.30	1.55 ± 0.54	3.20 ± 2.00
CD23^+^/B220^+^(×10^5^ cells)		20.03 ± 1.43	21.74 ± 5.95	19.52 ± 3.04	27.56 ± 5.35	18.15 ± 3.36	10.45 ± 1.45
CD4^+^(×10^5^ cells)	D-skin	10.93 ± 3.76	309.31 ± 36.62^###^	67.02 ± 15.85***	156.01 ± 21.98***	116.63 ± 21.21***	27.73 ± 1.81***
CD8^+^(×10^5^ cells)		42.08 ± 37.81	25.55 ± 17.65	62.02 ± 15.85	27.83 ± 20.84	24.03 ± 15.27	8.47 ± 4.65
Gr-1^+^/CD11b^+^(×10^5^ cells)		3.15 ± 1.17	78.18 ± 21.84^##^	12.49 ± 2.52**	25.20 ± 8.68**	15.34 ± 1.08**	4.87 ± 1.28***

Fluorescence-activated cell sorting analysis (FACS) of immune cell subtypes in ALN and spleen and D-skin. ^#^p < 0.05, ^##^p < 0.01, and ^###^p < 0.001 (compared with NC/Nga_Nr) and *p < 0.05, **p < 0.01, and ***p < 0.001 (compared with DNCB-CTL).

### The Effects of DHG on the Production of Th2 Cytokines (IL-4, IL-5, IL-13) and Th1 Cytokines (IFN-γ) by Cultured Splenocytes

**FIGURE 2:**
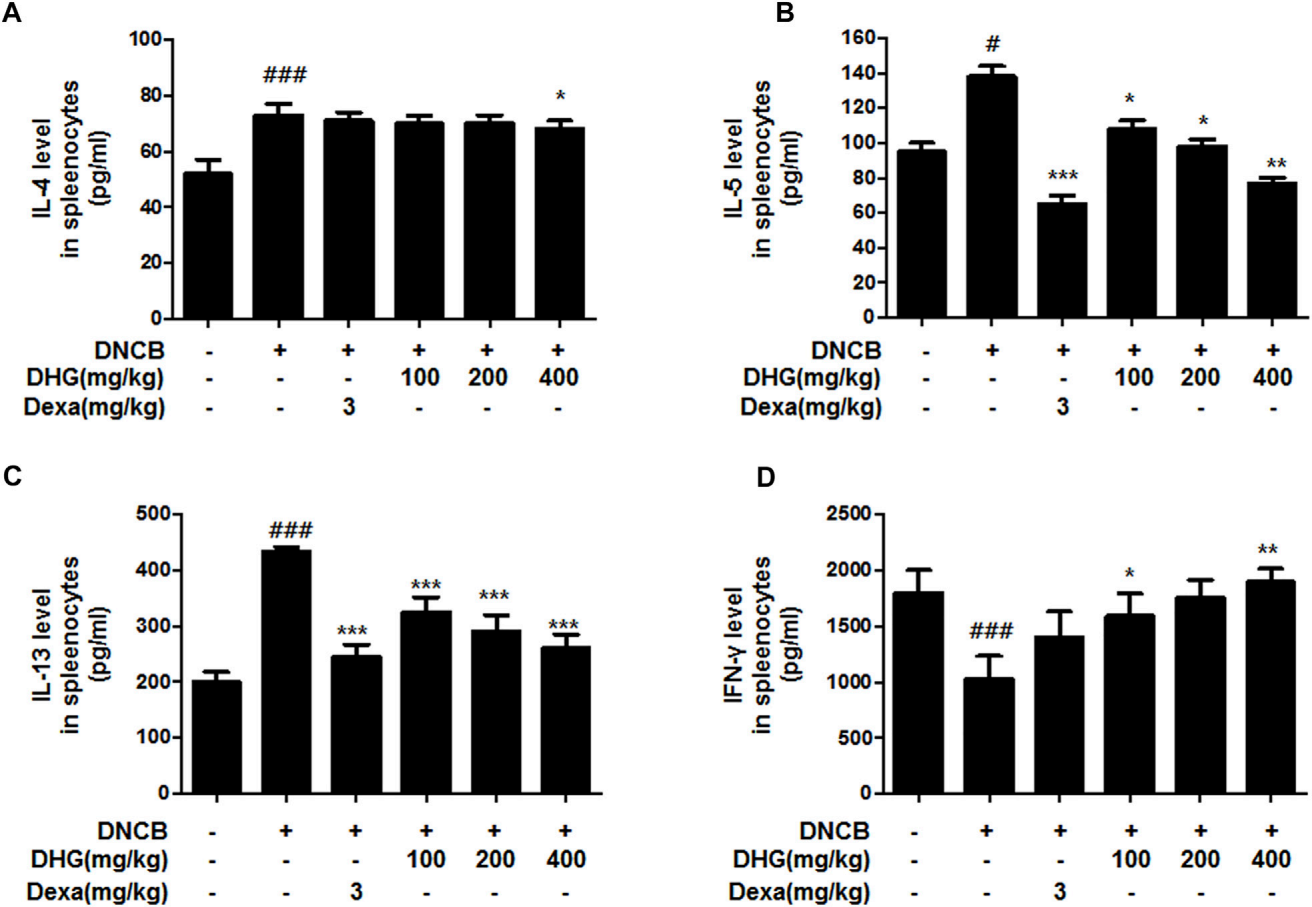
The effects of DHG on the production of IL-4 **(A)**, IL-5 **(B)**, IL-13 **(C)** and IFN-γ **(D)** by cultured splenocytes in NC/Nga mice (total *n* = 6/group). Splenocytes from mouse at 15 weeks of age were re-stimulated with CD3 mAb (0.5 μg/ml) for 48 h. IL-4, IFN-γ, IL-5, and IL-13 levels were measured by a sandwich ELISA. ^#^ *p* < 0.05, ^##^ *p* < 0.01, ^###^ *p* < 0.001 (compared with NC/Nga-Nr). *p < 0.05, **p < 0.01, and ***p < 0.001 (compared with DNCB-CTL).

In the culture supernatant of splenocytes, the DNCB_CTL group showed increase of IL-4, IL-5, IL-13 and decrease of IFN-γ to a statistically significant level (Figure 2), while the DNCB_Dexa group showed a statistically significant decrease in the IL-5 and IL-13 protein production (Figure 2B; Figure 2C). Among the experimental groups, the IL-4 protein production level was shown to have statistically significantly decreased in the DNCB_DHG 400 mg/kg group (Figure 2A). The IL-5 and IL-13 protein production also exhibited a trend of statistically significant decrease in the experimental groups (Figure 2B; Figure 2C), while IFN-γ protein production was statistically significantly higher in the DNCB_DHG 100 mg/kg and DNCB_DHG 400 mg/kg groups (Figure 2D). IL-5 and IFN-γ showed a tendency to decrease and increase in DNCB_DHG 200 mg/kg group compared to the DNCB_CTL group, while they did not show statistical significance.

### The Effects of DHG on mRNA Expression in Dorsal Skin Tissue

**FIGURE 3:**
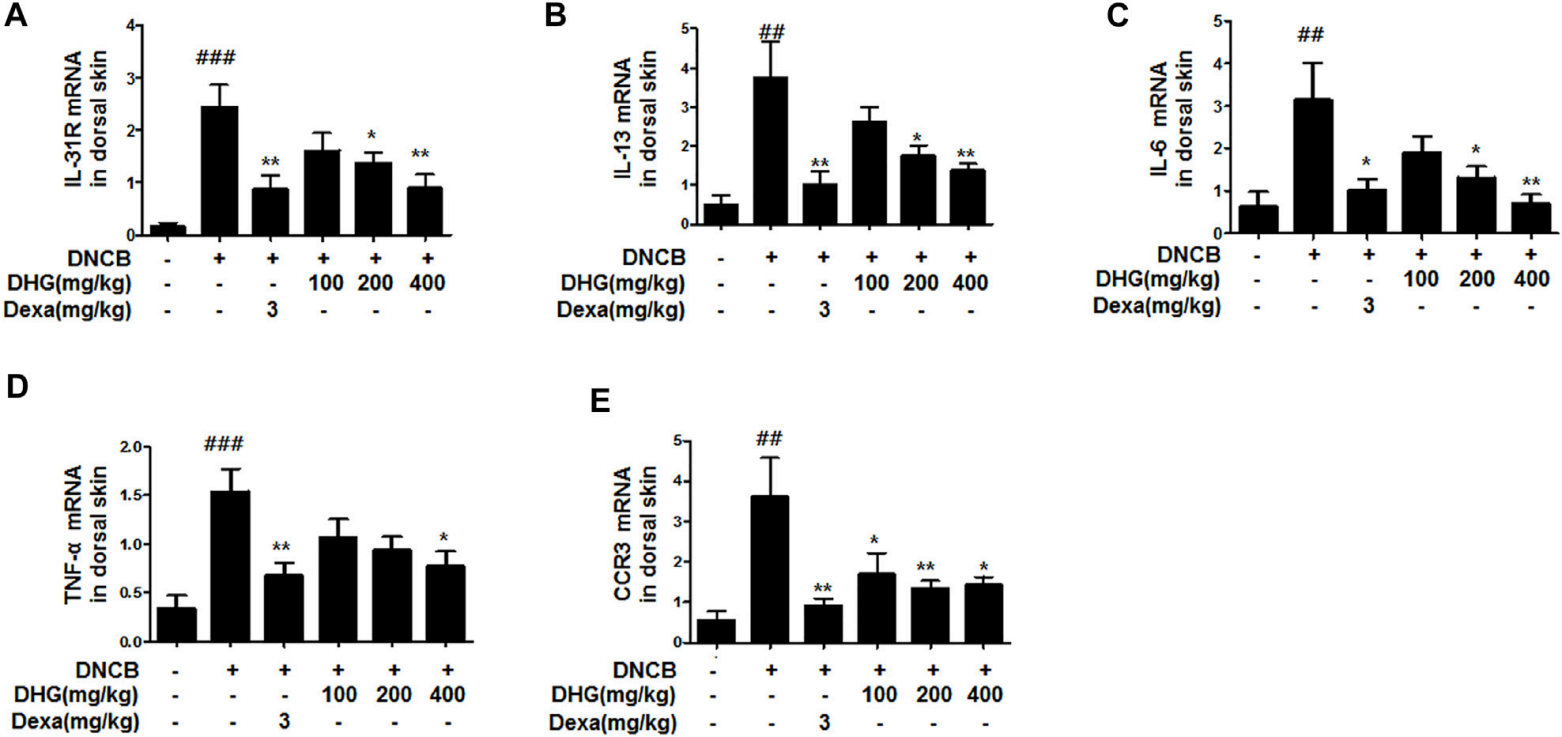
Effects of DHG on IL-31R **(A)**, IL-13 **(B)**, IL-6 **(C)**, TNF-α **(D)**, and CCR3 **(E)** and CCR3 mRNA expression in dorsal skin tissue (total *n* = 6/group). Total RNAs were extracted in dorsal skin tissue and IL-31R, IL-13, CCR3, IL-6, and TNF-α mRNA expression was analyzed by real-time PCR. The cycle number at which the emission intensity of the sample rises above the baseline is referred to as the RQ (relative quantitative) and is proportional to the target concentration. Real-time PCR was performed in duplicate and analyzed by Applied Biosystems 7,500 Fast Real-Time PCR system. ^#^ *p* < 0.05, ^##^ *p* < 0.01, ^###^ *p* < 0.001 (compared with NC/Nga-Nr). *p < 0.05, **p < 0.01, and ***p < 0.001 (compared with DNCB-CTL).

In the dorsal skin tissue, the relative quantitative (RQ) values of the mRNA expression of Th2 cell-related IL-31R, IL-13, IL-6, TNF-α, and CCR3 were statistically significantly higher in the DNCB_CTL group, whereas the DNCB_Dexa group showed a statistically significant decrease. Among the experimental groups, the DNCB_DHG 400 mg/kg group showed a statistically significant decrease that resembled the level shown by the DNCB_Dexa group, while the rate of reduction increased in a concentration-dependent manner (Figure 3).

### The Effects of DHG in Cell Frequency of Neutrophils, Eosinophils, and Lymphocytes in PBMCs Based on FACS Analysis

**FIGURE 4:**
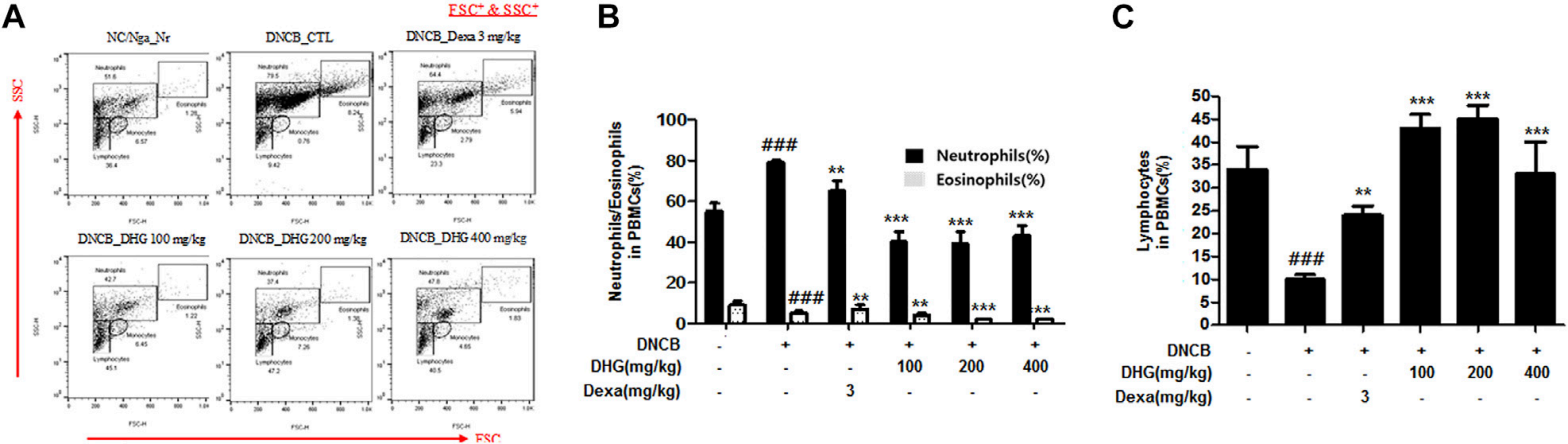
The effects of DHG on the percentage of neutrophils^,^ eosinophils, and lymphocytes changes of total cell content numbers in PBMCs in NC/Nga mice (total *n* = 6/group). Total cell content of FSC^+^ and SSC^+^ in PBMCs **(A)**. PBMC cells (5 × 10^5^ cells/mL) were isolated from blood and the PBMCs were washed twice and analyzed by flow cytometry **(B)**. ^#^ *p* < 0.05, ^##^ *p* < 0.01, ^###^ *p* < 0.001 (compared with NC/Nga-Nr). *p < 0.05, **p < 0.01, and ***p < 0.001 (compared with DNCB-CTL).

In the FACS analysis of PBMCs carried out at the end of the experiment, the total cell frequency of neutrophils and eosinophils showed a statistically significant increase in the DNCB_CTL group, while a statistically significant reduction was displayed by the experimental groups. The total cell frequency of lymphocytes showed a statistically significant decrease in the DNCB_CTL group, while a statistically significant increase was shown by experimental groups (Figure 4).

### The Effects of DHG on Serum IgE

**FIGURE 5:**
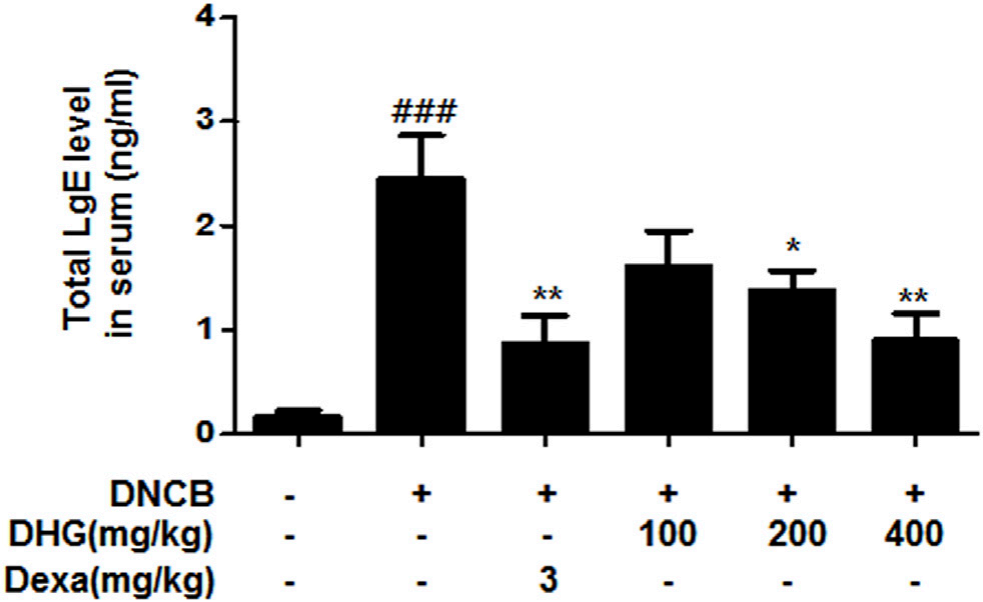
The effects of DHG on serum IgE level in NC/Nga mice (total n = 6/group). Total IgE levels were measured by a sandwich ELISA. ^#^ *p* < 0.05, ^##^ *p* < 0.01, ^###^ *p* < 0.001 (compared with NC/Nga-Nr). *p < 0.05, **p < 0.01, and ***p < 0.001 (compared with DNCB-CTL).

The IgE level in 15-week-old NC/Nga mice was significantly higher in the DNCB_CTL group, whereas the DNCB_Dexa group showed a statistically significant reduction. The IgE level in DNCB_DHG 200 mg/kg group and DNCB_DHG 400 mg/kg also showed decrease, while DNCB_DHG 400 mg/kg group did not show statistical significance (Figure 5).

### Dorsal Skin Tissue Examination and Analysis

**FIGURE 6:**
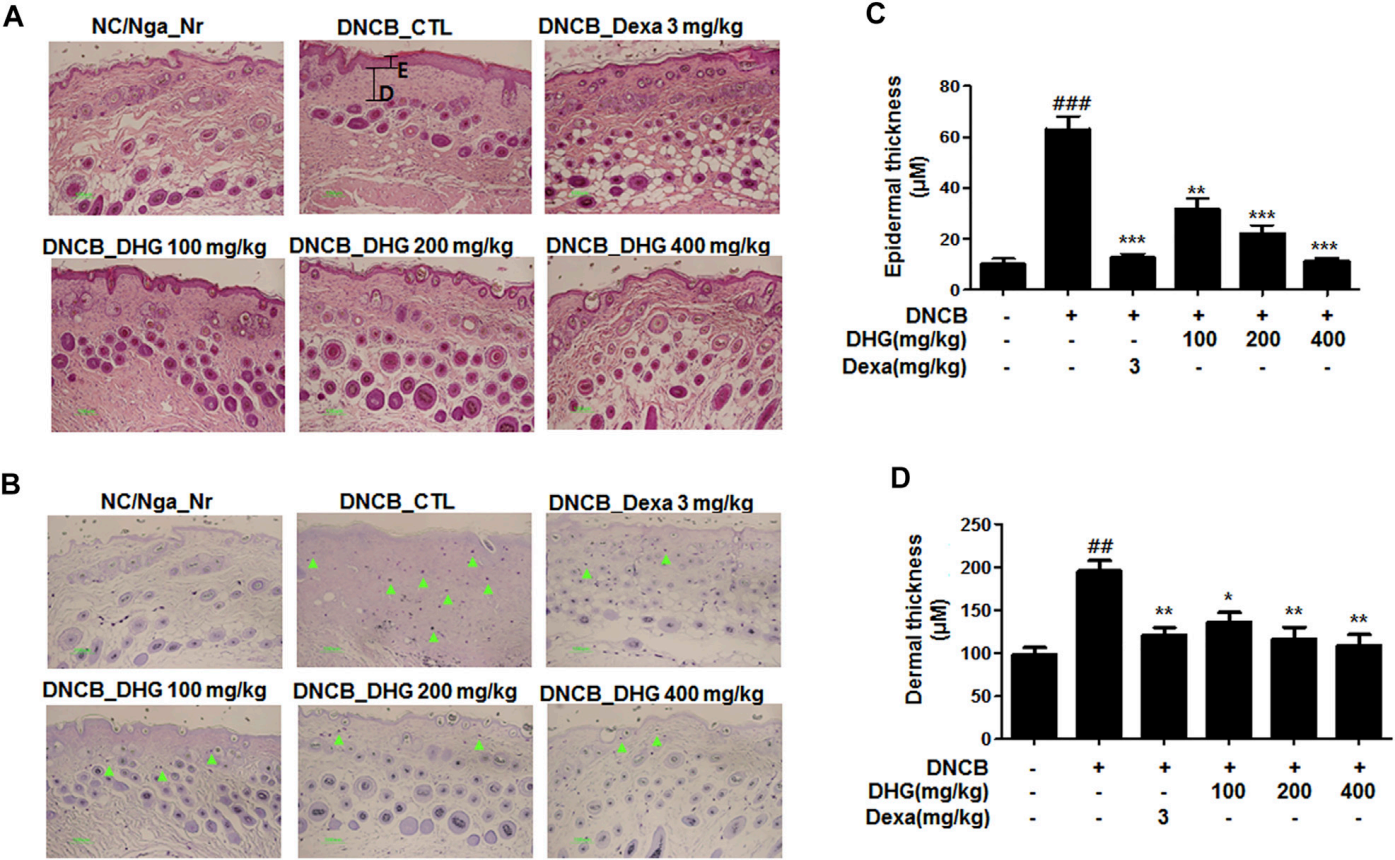
Histological features of dorsal skin tissue in NC/Nga mice (total *n* = 6/group). Skin biopsy were dyed with H and E **(A)** and showed the change of the epidermis by bright microscope (x200). Skin biopsy were dyed with toluidine blue **(B)** and exposed the degranulated mast cells by bright microscope (x200) Epidermal and dermal thickness **(C, D)** were measured in HE stained section (Black line).

Mast cell infiltration in the dermis, sections of dorsal skin were stained with H and E and toluidine blue (Figures 6A, B). The result showed that a significant level of hyperplasia and expansion of epidermal thickness was observed for the DNCB_CTL group, compared to the NC/Nga_Nr group, while the cells surrounding the tissue displayed significantly increased pigmentation, parakeratosis, mast cell infiltration (green arrow), and granulation production after the application of DNCB. The epidermal thickness in the DNCB_Dexa group and the experimental groups was substantially lower than the DNCB_CTL group, while some exhibited a level similar to the NC/Nga-Nr group. The keratosis, mast cell infiltration, and granulation production of the surrounding tissues also showed a significant reduction after the administration in the DNCB_Dexa group and the experimental groups.

### The Effects of DHG on TNF-α/IFN-γ–Induced Production of Pro-Inflammatory and Chemokines and the Expression of ICAM-1, COX-2 in HaCaT Cells

**FIGURE 7:**
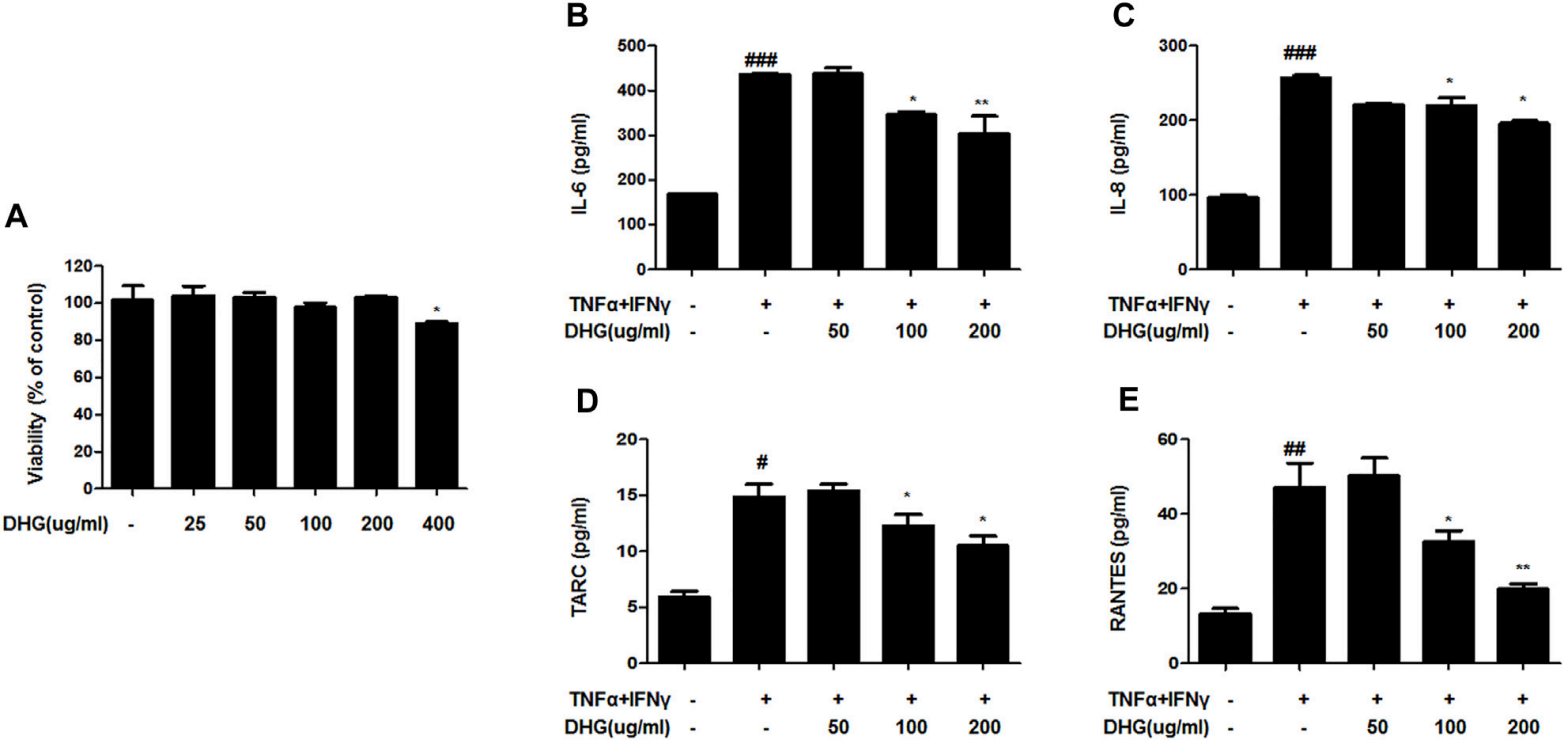
The effects of DHG on production of pro-inflammatory and chemokines in TNF-α/IFN-γ–induced HaCaT cells. The cytotoxicity of DHG on HaCaT cell **(A)**.The production of IL-6 **(B)**, IL-8 **(C)**, TARC **(D)**, RANTES **(E)**, were measured by using the culture supernatant of TNF-α/IFN-γ–stimulated HaCaT cells. The cells were pretreated with the indicated concentrations of DHG for 3 h and then stimulated with TNF-α/IFN-γ (each 10 ng/ml) for 24 h. Data are represented as mean ± SD of three independent experiments. *P < 0.05, **P < 0.01. compared with the TNF-α/IFN-γ-treated group.

**FIGURE 8:**
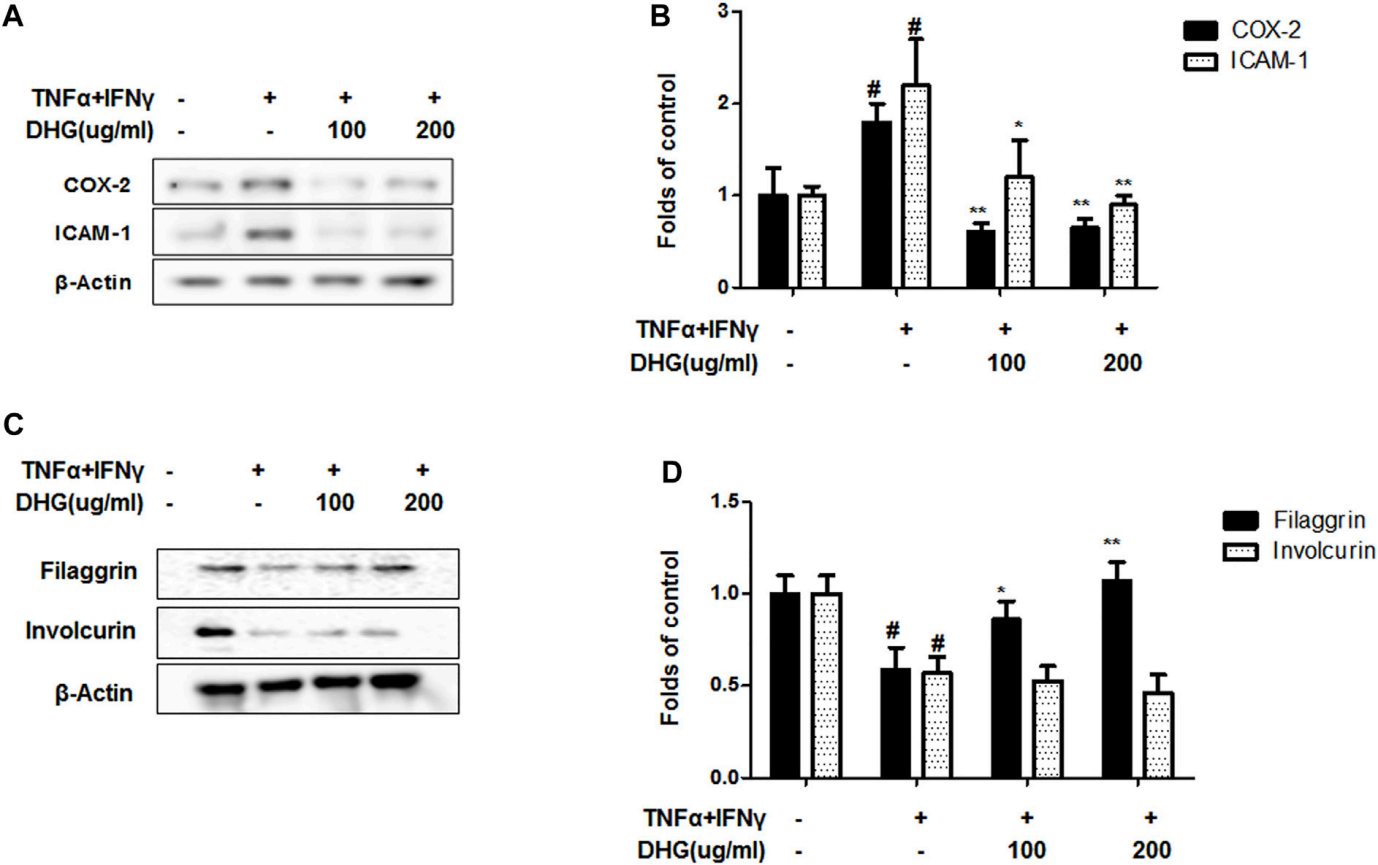
The effects of DHG on expression of COX-2/ICAM-1 **(A,B)** and Filaggrin/Involcurin **(C,D)** in TNF-α/IFN-γ–induced HaCaT cells. The expression of ICAM-1, COX-2, Filaggrin and Involcurin were measured by using cell lysis. The cells were pretreated with the indicated concentrations of DHG for 3 h and then stimulated with TNF-α/IFN-γ (each 10 ng/ml) for 24 h. Data are represented as mean ± SD of three independent experiments. *P < 0.05, **P < 0.01. compared with the TNF-α/IFN-γ-treated group.

To investigate the anti-AD effect of DHG on pro-inflammatory cytokines and chemokines production upon TNF-α/IFN-γ co-stimulation, HaCaT cells were pretreated with DHG for 3 h followed by TNF-α/IFN-γ for 24 h and the supernatant was collected for cytokine level measurement by ELISA (Figures 7A–D). The results showed that DHG inhibited the production of RANTES, IL-6, IL-8, and TARC. We also investigated the effects of DHG on ICAM-1and COX-2 expression in HaCaT cells, as shown in Figures 8A, B, DHG significantly reduced ICAM-1 and COX-2 expression levels compared with treatment with TNF-α/IFN-γ. Collectively, these results suggested that DHG significantly suppressed the production of pro-inflammatory and chemokines and the expression of ICAM-1, COX-2 in HaCaT cells.

### The Effects of DHG on Barrier-Related Protein, Filaggrin and Involcurin in HaCaT Cells

The distinctive features of AD are widespread barrier dysfunction. The barrier dysfunction correlates with the downregulation of barrier-related molecules such as Filaggrin and Involucrin. In inflammatory keratinocytes, the expression of these protein will be weakened (Masutaka, 2020) As showed in Figures 8C, D, TNF-α/IFN-γ group showed a decline of filaggrin and involcurin. DHG pre-treatment could up-regulated the expression of filaggrin, but DHG showed no effects on the expression of Involcurin.

### Effect of DHG on NF-κB and MAPK Signaling Pathways in TNF-α/IFN-γ-Stimulated HaCaT Cells

**FIGURE 9:**
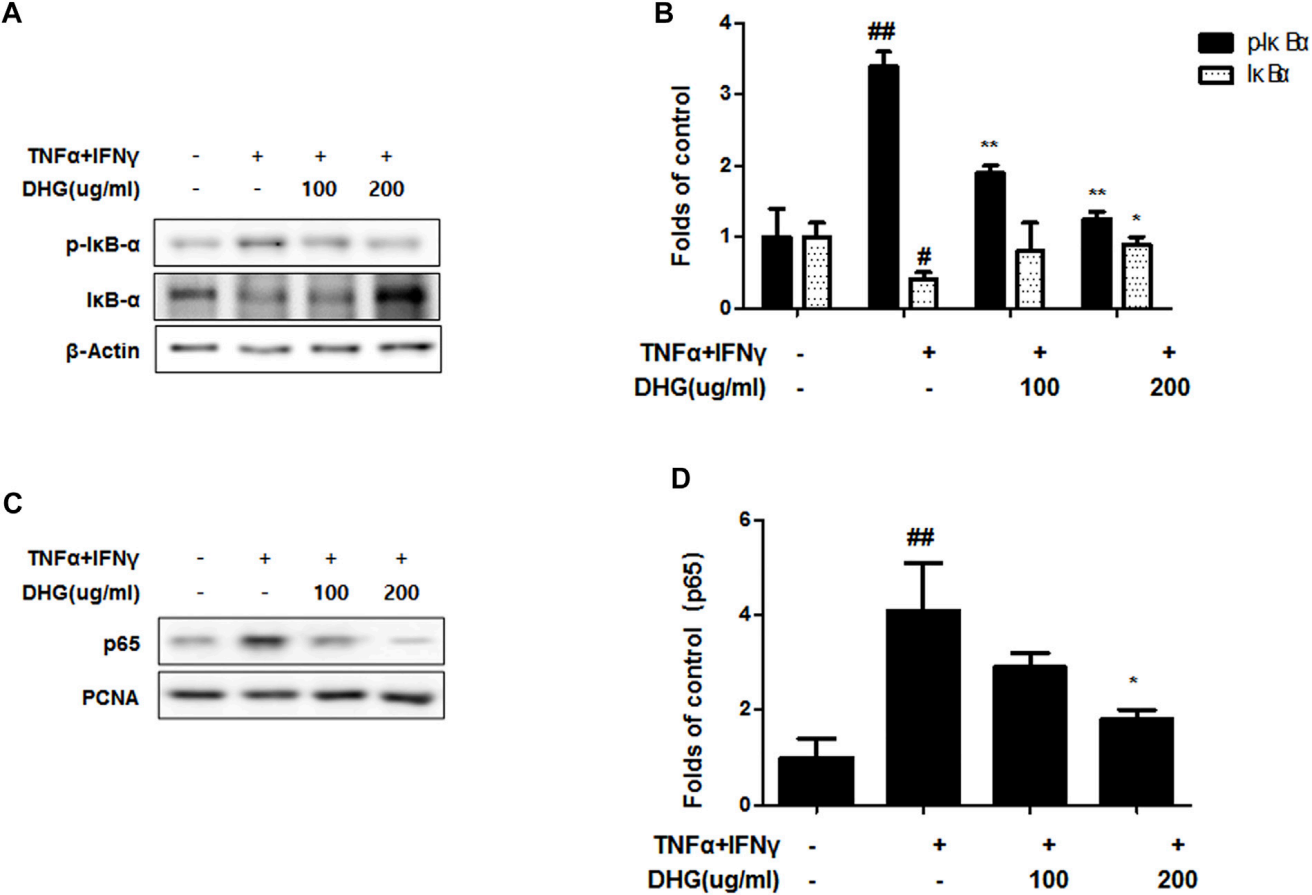
Effects of DHG on NF-κB activation **(A—D)** in TNF-α/IFN-γ stimulated HaCaT cells. The cells were pre-treated with the indicated concentrations of DHG for 3 h and then stimulated with TNF-α/IFN-γ (each 10 ng/ml) for 15 min. Cytosol and nuclear extracts were isolated, and levels of p65, p-IκBα and IκBα in fractions were determined by western blotting. Total proteins were prepared and were determined by western blotting. The bar graphs represent quantitative densities of the bands. Data are represented as mean ± SD of three independent experiments. *p < 0.05, **p < 0.01 compared with the TNF-α/IFN-γ-treated group.

**FIGURE 10:**
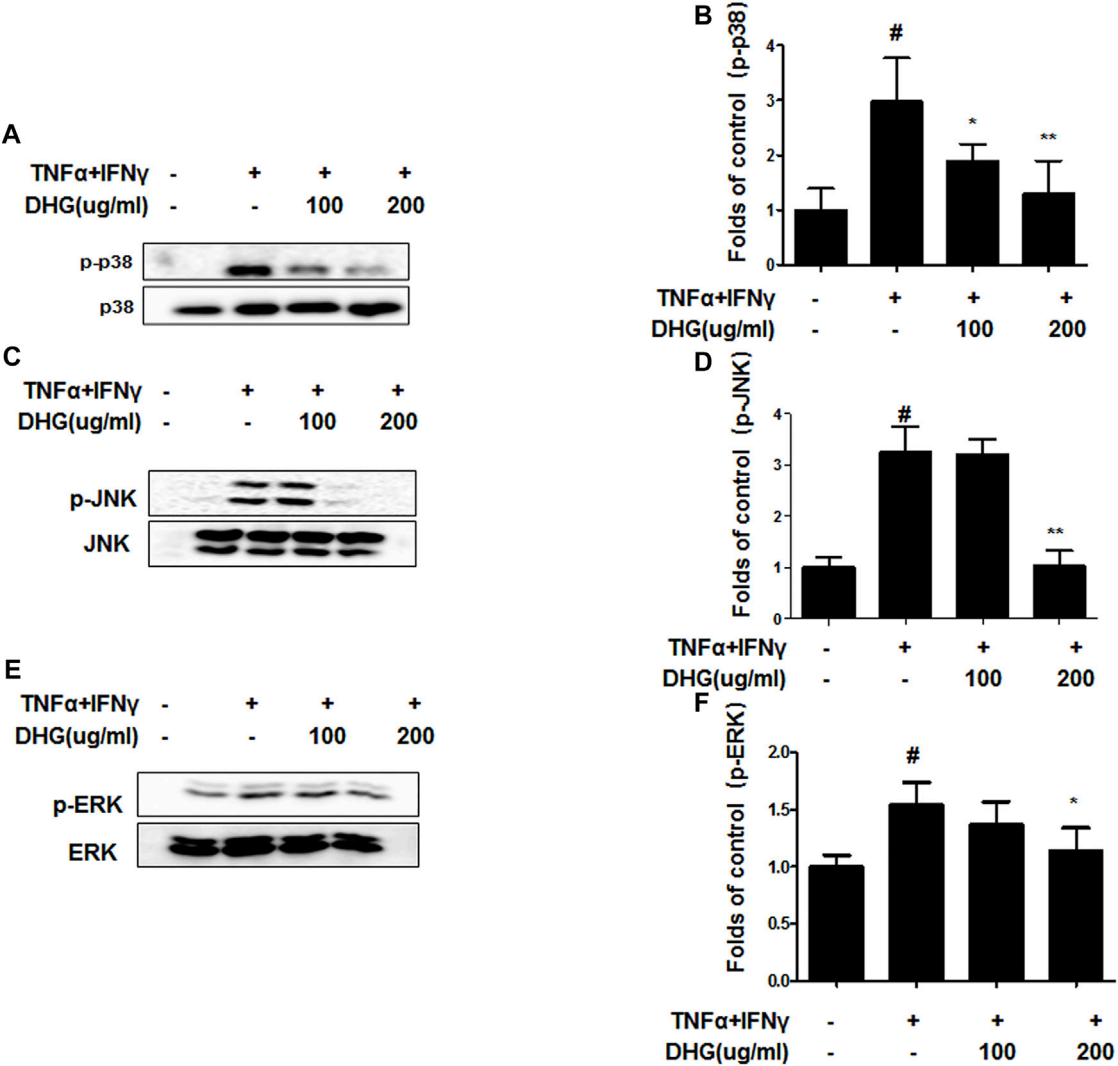
Effects of DHG on p-p38 **(A,B)**, p-JNK **(C,D)**, and p-ERK **(E,F)** MAPK activation in TNF-α/IFN-γ stimulated HaCaT cells. The cells were pre-treated with DHG for 3 h and then stimulated with TNF-α/IFN-γ for 1 h. Total proteins were prepared and were determined by western blotting. The bar graphs represent quantitative densities of the bands. Data are represented as mean ± SD of three independent experiments. *p < 0.05, **p < 0.01 compared with the TNF-α/IFN-γ-treated group.

We investigated the effects of DHG on the expression of inflammation-related factors such as MAPK and NF-κB-p65 in TNF-α/IFN-γ-stimulated HaCaT cells. As shown in Figure 9, DHG inhibited TNF-α/IFN-γ-induced NF-κB p65 phosphorylation, IκBα phosphorylation, and degradation. In addition, we also checked the effects of DHG on MAPK activities such as p38, extracellular signal-regulated kinases (ERK), and c-Jun N-terminal kinases (JNK) in Figure 10. The relative abundances of proteins were calculated for the p-ERK/ERK, p-p38/p38, and p-JNK/JNK ratios. As a result, DHG strongly inhibited the activation of p38, JNK and ERK induced by TNF-α/IFN-γ.

## Discussion

The onset of atopic dermatitis is due to an antigen disturbing the balance of Th1/Th2 cytokines to cause overproduction of Th2 cytokines such as IL-4, IL-5, and IL-13, while stimulating the B cells to increase the serum IgE level (Leung et al, 2004). The increased IgE binds to the receptors on the surface of mast cells for desensitization, and the antigen would bind with the IgE on the mast cell surface upon reinvasion to induce mast cell activation and degranulation. During the process of degranulation, v transmitters such as histamine are released to the cell surface, whereby the symptoms of allergy appear (Prussian and Melcafe, 2003). In the acute phase of atopic dermatitis, Th2 cells develop preferentially, but as the symptoms aggravate, Th1 cytokines show simultaneous increase to further accelerate the inflammatory response (Oyoshi et al., 2009). During this process, Th2 cells produce various cytokines and chemokines to facilitate the progression of atopic dermatitis. IL-4, IL-5, and IL-13, the cytokines expressed in Th2 cells, induce the production and differentiation of B lymphocytes (Smith et al., 2000). IL-4 facilitates B cell activation and the production of MHC class II (Stuart et al., 1988). IL-5 is produced in Th2 cells, to play a critical role in eosinophil development, survival, and activation (Simon et al., 2004). IL-13 performs a role of inhibiting the macrophage activity to destroy microorganisms (Doherty et al., 1993). IFN-γ increases the antimicrobial activity of macrophages, while upregulating the expression of the main regulators that promote the Th1 lymphocyte differentiation and activation, and inhibit the expansion of the Th2 group (Gajewski and Fitch, 1988; Sibley et al., 1988; Mosmann and Sad, 1996). The skin lesions in atopic dermatitis patients contain a greater number of Langerhans cells and cell surface receptors that deliver allergens, compared to healthy individuals (Olivry et al., 1996). The Langerhans cells in skin lesions are able to mediate powerful stimulation of T-lymphocytes, and the antigen-specific IgE increases such ability of Langerhans cells in antigen delivery (Olivry et al., 1996). Langerhans cells deliver IgE-bound antigens to the lymphocytes, thereby playing a role of inducing and sustaining the cell-mediated immune responses (Dubra et al., 2010).

Human keratinocytes-HaCaT cells, are one of the cell lines used to mimic AD-like inflammatory symptoms *in vitro* in response to inflammatory stimulations, such as TNF-α and IFN-γ. HaCaT cells release pro-inflammatory cytokines, chemokines, and proteases after TNF-α/IFN-γ stimulation and contribute to inflammation and immune responses. In particular, keratinocyte-derived TARC/CCL17, MDC/CCL22, and RANTES/CCL5 play a major role in AD initiation. In addition, IL-6, IL-8 is an important cytokine that activates chemotaxis in neutrophils and granulocytes, which then migrate toward the infection site. In this study, DHG dose-dependently inhibited the production of pro-inflammatory cytokines IL-6 and IL-8 and chemokines TARC and RANTES in TNF-α/IFN-γ-induced HaCaT cells, as well as the expression of ICAM-1 and COX-2. We believe that DHG has potential for application as an anti-AD agent. TNF-α/IFN-γ induces keratinocyte inflammation via modulating different signaling pathways including the mitogen-activated protein kinases (MAPKs) and the nuclear factor-κB (NF-κB) pathways (Ngo et al., 2020).

DHG is a solid, gelatinous substance that has been widely used in the form of foods, crafts, and drug in East Asia such as China and Korea. DHG is composed of amino acids, proteins/gelatin, polysaccharides, volatile substances, minerals (Wang et al., 2014; Kang et al., 2022) and can replenish the amino acids to the damaged skin tissues in the body, with an ability to promote skin regeneration and recovery (Chen et al., 1991; Tian et al., 2017; Lee et al., 2010). It has been reported that DHG have specific peptides, such as PEP-1 and PEP-2, which have been reported that binding with FK506 to enhance the anti-inflammatory activity (Breiman and Camus 2002). FK506 binding proteins belong to a family of immunophilins that were named for their ability bind immunosuppressive drugs. PEP-1-FK506BP can significantly inhibit alkali burn-induced corneal inflammation in rats, possibly by accelerating corneal wound healing and by reducing the production of angiogenic factors and inflammatory cytokines (Kim et al., 2015) what’ more, Topical application of PEP-1-FK506BP to NC/Nga mice markedly inhibited AD-like dermatitis (Kim et al., 2011). This suggests that PEP-1-FK506BP could be used as a potential therapeutic agent for inflammatory diseases such as AD (Kim et al., 2011a; Kim et al., 2011b). Our previous paper is the confirming for the inhibitory effect of house dust mite (HDM) allergens among the atopic dermatitis (AD) models and three types of administration routes with DHG, to determine the effective application of DHG (Kang et al., 2022). This study was designed to show that DHG is able to concentration-dependently alleviate atopic dermatitis by inducing anti-inflammatory and anti-allergic activities through the regulation of immune-related substances in the body. In addition, the *in vitro* study also identified factors related to skin inflammation and skin barrier protection.

In this study, compared to the NC/Nga_Nr group, the DNCB_CTL group where atopic dermatitis was induced by DNCB, showed statistically significant increase in total cell count, neutrophil and eosinophil cell frequency, and various immune substances. In H and E and toluidine blue staining assays at the end of the experiment, the DNCB_CTL group, compared to the NC/Nga_Nr group, showed significantly higher levels of hyperplasia and expansion of epidermal thickness and of inflammatory and allergic reactions in the areas surrounding the tissue. In the FACS analysis, the experimental groups in comparison to the DNCB_CTL group showed a statistically significant reduction in the absolute number of CD3^+^, CD4^+^, CD4^+^/CD69 and CD23^+^/B220^+^ in ALN, and of CD4^+^ and Gr-1^+^/CD11b^+^ in dorsal skin tissue. In addition, the relative quantitative (RQ) values of IL-31R, IL-13, CCR3, TNF-α and IL-6 mRNA expressions related to Th2 cells collected from the dorsal skin tissue, showed a significant reduction in the DNCB_DHG 400 mg/kg group to the level of the DNCB_Dexa group. This indicated that the administration of DHG could inhibit the mRNA expression of the Th2-related immune substances that have an influence on allergy, in a concentration-dependent manner. In this study, we also investigated the early signaling pathways of TNF-α/IFN-γ induced keratinocytes, the MAPK family includes ERK, JNK, and p38, which control a variety of physiological process. In our results, we found that DHG could inhibit the expression of p-ERK, p-JNK and p-p38 induced by TNF-α/IFN-γ. On the other hand, NF-κB is well known as a transcription factor that regulates many immune and inflammatory responses. NF-κB combines with IκBα in cytoplasm in normal condition, some stimulation such as TNF-α/IFN-γ could cause the disaggregation of the IKK complex, the IκBα phosphorylates leading the proteasomal degradation of IκBα. The NF-κB could translocated to the nucleus to activates target genes. In our study, TNF-α/IFN-γ could activate the NF-κB signaling pathway, it manifested as the increase of p-IκBα, the translocation of NF-κB from cytoplasm to nucleus and the degradation of IκBα. Our results showed that DHG could inhibit the translocation of NF-κB, increase the level of IκBα and reduced the phosphorylation of IκBα.

In the analysis involving the spleen cell culture solution, the levels of IL-4, IL-5, and IL-13 protein production showed a statistically significant reduction in the DNCB_DHG 400 mg/kg group, compared to the DNCB_CTL group, whereby the production resembled the level in the DNCB_Dexa group. The protein production of IFN-γ group, on the contrary, showed a statistically significant increase in the DNCB_DHG 100 mg/kg and DNCB_DHG 400 mg/kg groups, compared to the DNCB_CTL group. A decrease in IFN-γ production in the peripheral blood and skin tissues of acute atopic dermatitis patients has been reported, and taking into consideration the fact that IFN-γ inhibits IgE production and Th2 lymphocyte proliferation (Renz et al., 1992; Leung et al., 2004; Simon D et al., 2004), the DHG water extracts are thought to suppress the skin inflammatory responses by inhibiting the Th2 cytokine production and regulating the IFN-γ expression.

## Conclusion

*In vivo* Nc/Nga mice experiments, the oral administration of Donkey Hide Gelatin (DHG) water extracts led to a statistically significant decrease in CD3^+^. CD4^+^, CD4^+^/CD69^+^, CD23^+^/B220^+^, and Gr-1^+^/CD11b^+^ absolute numbers and the mRNA expression of Th2 cytokines, such as IL-31R, IL-13, CCR3, TNF-α, and IL-6 in ALN and dorsal skin tissue. DHG increased the level of IFN-γ in cultured splenocytes while the reducing the IL-4, IL-5, and IL-13 protein production levels. Through this, the levels of neutrophils and eosinophils and serum IgE decreased, and the allergy-induced inflammatory reactions were alleviated. In the histology analysis of the dorsal skin tissue of the experimental groups, the epidermal thickness was significantly reduced, accompanied by a significant level of reduction in hyperkeratinization, parakeratosis, pigmentation, mast cell infiltration and granulation in the areas surrounding the tissue. *In vitro* experiments of HaCaT cell, DHG significantly suppressed the production of pro-inflammatory and chemokines such as RANTES, IL-6, IL-8, TARC and the expression of ICAM-1, COX-2. What’ more, DHG could up-regulated the expression of Filaggrin which may exerts beneficial effects in AD. The mechanism underlying the anti-inflammatory activity of DHG may be through MAPK-p38 and ERK and NF-κB signaling pathway. Taken together, this study suggests that DHG could be a potential therapeutic agent to prevent and cure atopic dermatitis.

## Data Availability

The datasets presented in this study can be found in online repositories. The names of the repository/repositories and accession number(s) can be found in the article/Supplementary Material.

## Abbreviations

TNF-α, tumor necrosis factor-α; IFN-γ, interferon-γ; DHG, Donkey Hide Gelatin; AD, Atopic dermatitis; MAPK, mitogen-activated protein kinases; DNCB, dinitrochlorobenzene; H and E, hematoxyline and eosin; GAPDH, glyceraldehyde-3 phosphate dehydrogenase; FACS, Fluorescence-activated cell sorting analysis; ERK, extracellular signal-regulated kinases; JNK, c-Jun N-terminal kinases; NF‐κB, nuclear factor‐κB; RQ, relative quantitative.
